# Efficacy of Dupilumab on Different Phenotypes of Adult with Moderate-to-Severe Atopic Dermatitis in Taiwan: A Real-World Study

**DOI:** 10.3390/jcm11206209

**Published:** 2022-10-21

**Authors:** Chin-Yi Yang, Po-Ju Lai, Chun-Bing Chen, Tom C. Chan, Rosaline Chung-Yee Hui, Yu-Huei Huang, Han-Chi Tseng, Shang-Hung Lin, Chun-Wei Lu, Hua-En Lee, Jing-Yi Lin, Min-Hui Chi, Ming-Feng Tsai, Yih-Shiou Hwang, Chuang-Wei Wang, Chia-Yu Chu, Wen-Hung Chung

**Affiliations:** 1 Department of Dermatology, New Taipei Municipal TuCheng Hospital, New Taipei City 236, Taiwan; 2 Department of Dermatology, Drug Hypersensitivity Clinical and Research Center, Linkou Chang Gung Memorial Hospital, Taoyuan 333, Taiwan; 3 School of Medicine, College of Medicine, Chang Gung University, Taoyuan 333, Taiwan; 4 Chang Gung Immunology Consortium, Linkou Chang Gung Memorial Hospital, Taoyuan 333, Taiwan; 5 Department of Dermatology, Chung Shan Medical University Hospital, Taichung 402, Taiwan; 6 Institute of Medicine, Chung Shan Medical University, Taichung 402, Taiwan; 7 Whole-Genome Research Core Laboratory of Human Diseases, Keelung Chang Gung Memorial Hospital, Keelung 204, Taiwan; 8 Genomic Medicine Core Laboratory, Linkou Chang Gung Memorial Hospital, Taoyuan 333, Taiwan; 9 Immune-Oncology Center of Excellence, Linkou Chang Gung Memorial Hospital, Taoyuan 333, Taiwan; 10 Department of Dermatology, Xiamen Chang Gung Hospital, Xiamen 361028, China; 11 School of Medicine, National Tsing Hua University, Hsinchu 300044, Taiwan; 12 Department of Dermatology, National Taiwan University Hospital, Taipei 100, Taiwan; 13 Department of Dermatology, National Taiwan University College of Medicine, Taipei 100, Taiwan; 14 Department of Dermatology, Drug Hypersensitivity Clinical and Research Center, Keelung Chang Gung Memorial Hospital, Keelung 204, Taiwan; 15 Department of Dermatology, Kaohsiung Chang Gung Memorial Hospital, Kaohsiung 833, Taiwan; 16 Graduate Institute of Clinical Medical Sciences, College of Medicine, Chang Gung University, Taoyuan 333, Taiwan; 17 Institute of Molecular Medicine, College of Medicine, National Taiwan University, Taipei 106, Taiwan; 18 Division of Plastic Surgery, Department of Surgery, Mackay Memorial Hospital, Taipei 104, Taiwan; 19 Department of Medicine, MacKay Medical College, New Taipei 252, Taiwan; 20 Graduate Institute of Biomedical Informatics, Collage of Medical Science and Technology, Taipei Medical University, Taipei 110, Taiwan; 21 Department of Ophthalmology, Linkou Medical Center, Linkou Chang Gung Memorial Hospital, Taoyuan 333, Taiwan; 22 Department of Ophthalmology, Jen-Ai Hospital Dali Branch, Taichung 412, Taiwan; 23 Department of Ophthalmology, Xiamen Chang Gung Memorial Hospital, Xiamen 361028, China

**Keywords:** atopic dermatitis, biomarker, dupilumab, eczema phenotype, efficacy

## Abstract

To determine phenotype-related dupilumab response in adult patients with atopic dermatitis (AD), this multicenter, retrospective study included 111 adults with moderate-to-severe AD in Taiwan, with median age of 31.5 years (18–87) and 71 (64.0%) males. Patients received dupilumab 300 mg per two to three weeks up to 12 months. We found a significant improvement after 4 and 16 weeks of treatment in all patients for all the assessed scores, including eczema area and severity index (EASI) improvement ≥50% (EASI-50) and 75% (EASI-75), EASI reaching minimal clinically important difference (MCID), and Investigator’s Global Assessment (IGA) improvement ≥2. Importantly, prior to asthma, early AD onset and 3-week drug intervals were significantly associated with a high proportion of EASI-75 at month 12, while prurigo and lichenoid phenotypes were associated with a lower proportion of EASI-75 at month 12. However, the majority of adverse events were mild in severity. In conclusion, our study results identify phenotype-related dupilumab response at month 12 in adults with moderate-to-severe AD, and we suggest that treatment should not be discontinued until reaching a satisfactory clinical response.

## 1. Introduction

Atopic dermatitis (AD) is a common chronic skin condition with a worldwide prevalence of 1% to 20% in adults, and approximately 20% of patients have moderate-to-severe disease [1,2,3]. It is characterized by T-helper (Th)-2-mediated skin inflammation and epidermal and barrier dysfunction [4]. The clinical presentation and severity of AD varies widely, and diagnosis is not always straightforward, especially in adults [5]. Despite the high prevalence of AD, effective treatments are limited, especially for patients with moderate to severe disease. Several systemic therapeutic options are approved for patients with severe disease who are not controlled by topical medications, including oral corticosteroids, oral cyclosporin, and UVA/narrow-band UVB phototherapy [6]. However, these treatments often have limited efficacy and unfavorable safety. Thus, a significant need remains for more effective and safe therapies.

Dupilumab was the first biological drug approved by both the US Food and Drug Administration (FDA) and the European Medicines Agency (EMA) for the treatment of adults with moderate-to-severe AD, and trials are underway to assess its safety as well as its effectiveness in the pediatric population [7]. Dupilumab is a human monoclonal antibody (mAb) binding to the shared alpha subunit of IL-4 and IL-13 receptors, and it modulates the activation of T cells through the IL-4 and IL-13 pathways. The receptor is additionally expressed on dendritic cells, keratinocytes, or eosinophils. In vitro studies showed that dupilumab induced a dose-dependent improvement of the molecular signature in AD skin. The suppression of mRNA expression of genes involved in the activation of T cells, dendritic cells or eosinophils has been observed [8]. Early clinical trials of dupilumab have shown clinical improvement in adults with moderate-to-severe AD [9,10,11]. The effect of dupilumab on AD has recently been investigated in two large phase-III trials [10]. Both trials included adults with AD that was inadequately controlled by topical treatment. Patients were treated over a period of 16 weeks with placebo or with 300 mg dupilumab administered weekly or in 2-week intervals. The primary endpoint of both trials was the Investigator’s Global Assessment (IGA) scale, which is validated scoring system for AD severity. Both studies had similar results and showed a reduction of 2 points or more in IGA at week 16 in 38%/36% of patients treated with dupilumab every 2 weeks and in 37%/36% of patients treated with dupilumab in weekly intervals. In both trials, a 75% reduction in the eczema area and severity index (EASI) was found in patients treated with dupilumab following one of two dosing regimens [10]. 

AD is a complex and multifactorial disease with a significant impact on the quality of life of afflicted patients and their families. Many of the uncertainties about AD are related to the striking heterogeneity of the disease, which is exemplified by variations in clinical features, disease course, and individual risk factors, and these are shown in the absence of reliable biomarkers, diagnostic tests, or specific treatments that would apply to all AD patients. Despite the unmet need for well tolerated, effective, and personalized treatment of AD, the current standard treatments for AD do not focus on individual phenotypes of the disease. The development of targeted, phenotype-specific therapies has the potential to open a promising new era of individualized treatment for AD. In that context, biomarkers would help to define AD phenotypes in order to identify potential targets for new therapeutic strategies. Therefore, the present study aimed to investigate associations between biomarker-based phenotypes and the efficacy of dupilumab in adult patients with moderate-to-severe AD. 

## 2. Materials and Methods 

### 2.1. Study Design and Sample 

This retrospective, multicenter (National University Hospital, Chang Gung Memorial Hospital, and Chung Shan Memorial Hospital) cohort study recruited adult (age ≥ 18 years) patients with moderate-to-severe AD who were treated with dupilumab from August 2018 to February 2021 in one of the three hospitals. Consecutive patients (*n =* 111), each with an observation period of at least 16 weeks, were enrolled as the analytic sample. 

Diagnosis of AD was made by an expert, board-certified dermatologist according to the Hanifin criteria, which is the diagnostic standard for AD [12]. These treatment criteria were established for patient enrollment in the dupilumab drug prescription appropriateness according to the Taiwan Medical Agency. Dupilumab was administered subcutaneously at label dosage (300 mg every 2 weeks). 

### 2.2. Ethical Considerations

Approval of the study protocol was obtained from the Internal Review Board (IRB) of Chang Gung Memorial Hospital (No. 202200642B0). Signed informed consent by patients was waived by the IRB because of the retrospective nature of the study.

### 2.3. Data Collection and Main Outcomes 

The following data were collected from patients’ medical records: demographic variables, Investigator’s Global Assessment (IGA) scores, body surface area (BSA), specific locations of lesions (face, hands, genitals), atopic/allergic conditions, previous treatments, changes in objective and subjective disease parameters and adverse events. Laboratory index, including eosinophil count, total immunoglobulin E (IgE), lactate dehydrogenase (LDH), and creatinine were also collected at baseline and after 1, 4 and 12 weeks of treatment. Disease severity was assessed at baseline and after 1, 4 and 12 weeks of treatment using the EASI score.

Patients were stratified into 7 clinical phenotypes (Figure S1) by describing the specific characteristics of adult AD: [5,13] (1) Type I, multiple extensive erythematous exudative and lichenoid plaques with flexor side of four limbs and trunk, head and neck; (2) Type II, generalized eczema with diffuse erythema, predominantly exudative, and crusted eczematous lesions; (3) Type III, prurigo with highly pruriginous papules and nodules; (4) Type IV, erythroderma with over 90% of the skin surface being red, dry, and lichenified; (5) Type V, nummular eczema with round, inflamed sores (mainly in, four limbs extensor sides); (6) Type VI, lichenoid pattern with generalized lichenification, excoriations, crusts, and xerosis; and (7) Type VII, pattern of follicular eczema.

Primary outcomes included percentage change of patients with ≥75% improvement in the EASI score from baseline (EASI-75). Secondary outcomes were percentage change of patients with ≥50% improvement or patients with ≥6.6 change in EASI from baseline (EASI-50 and minimal clinically important difference (MCID), [14] respectively).

### 2.4. Statistical Analysis

Descriptive statistics are presented as median (range) or proportion (%). Categorical variables were classified into two groups, above or below the median. Eosinophilia was defined as >500 cells/mm^3^. Intrinsic AD was considered IgE < 100 kU/L. The effectiveness of treatment was evaluated at month 1, 4, and 12 in terms of percentage change from baseline and percentage of patients with an EASI reduction >50% and >75% from baseline (EASI-50, EASI-75). The effectiveness of treatment was evaluated at months 4 and 12 in terms of IGA < 2 at month 4 and 12. The Wilcoxon matched-pairs rank test was used to evaluate differences between baseline and follow-up. Fisher’s exact test was used to test associations between patients’ characteristics and EASI-75 at months 4 or 12. Statistical analyses were performed by IBM-SPSS software v.25 (IBM SPSS, Chicago, IL, USA). Differences were considered significant at *p* < 0.05.

## 3. Results

### 3.1. Patient Characteristics

A total of 111 adult patients, 40 women and 71 men, who were diagnosed as moderate-to-severe AD and treated with dupilumab were included (Table 1). Median age at onset was 18 years (0–72), and median age at dupilumab initiation was 30 (18–79). Prior allergic rhinitis and asthma were found in 78/111 (70.3%) and 29/111 (26.1%) patients, respectively. Renal insufficiency, liver disease, and hyper-IgE syndrome were observed in five (4.5%), four (3.6%), and two (1.8%) patients, respectively. Adult-onset AD was observed in 52/100 (52.0%) patients, and an intrinsic type (baseline total IgE < 100) of AD was observed in 2/65 (3.1%) patients. A high proportion of the AD phenotype (50/99, 50.5%) displayed multiple extensive erythematous exudative and lichenoid plaques with flexor side of four limbs and trunk, head and neck. Previous treatment with cyclosporine, azathioprine, or methotrexate was noted in 52/111 (46.8%) patients (Table 1). All patients were treated with 300 mg dupilumab every second week. Of these, 66 (59.5%) patients received drug interval change of dupilumab after 4 to 6 months of treatment, including 22 patients who received an interval change of 3 weeks, 31 patients who received an interval change of 1 month, 8 patients who received an interval change of 2 months, and 1 patient who received an interval change of 3 months.

### 3.2. Efficacy of Dupilumab Treatment

Table 2 displays the number of patients who reached 1 month of treatment (111 patients), 4 months of treatment (111 patients), and 12 months of treatment (47 patients). The reduction in the number of patients who reached 12 months of treatment was because patients started treatment at different times and all patients did not reach 12 months of treatment by the time the present study was published. A very low percentage of patients discontinued treatment (18 out of 111 patients, Table S1). Table 2 shows the assessment of EASI-50 (50% reduction in EASI score from baseline), EASI-75 (75% reduction in EASI score from baseline), IGA score, and EASI reaching MCID (improvement in EASI score from baseline ≥6.6) at 1, 4, or 12 months of treatment of all patients. After 1 month of treatment, 18 (16.2%) patients had a response of EASI-75, 41 (36.9%) patients had a response of EASI-50, and the EASI score reached MCID in 72 (64.9) patients. After 4 months of treatment, 49 (44.1%) patients had a response of EASI-75, 96 (86.5%) patients had a response of EASI-50, 29/106 (27.4%) patients had an IGA score <2, and EASI score reached MCID in 104 (93.7%) patients. Lastly, after 12 months of treatment, 29/47 (61.7%) patients had a response of EASI-75, 46/47 (97.9%) patients had a response of EASI-50, 23/51 (45.1%) patients had an IGA score <2, and the EASI score reached MCID in 47/47 (100%) patients. 

**Figure 1:**
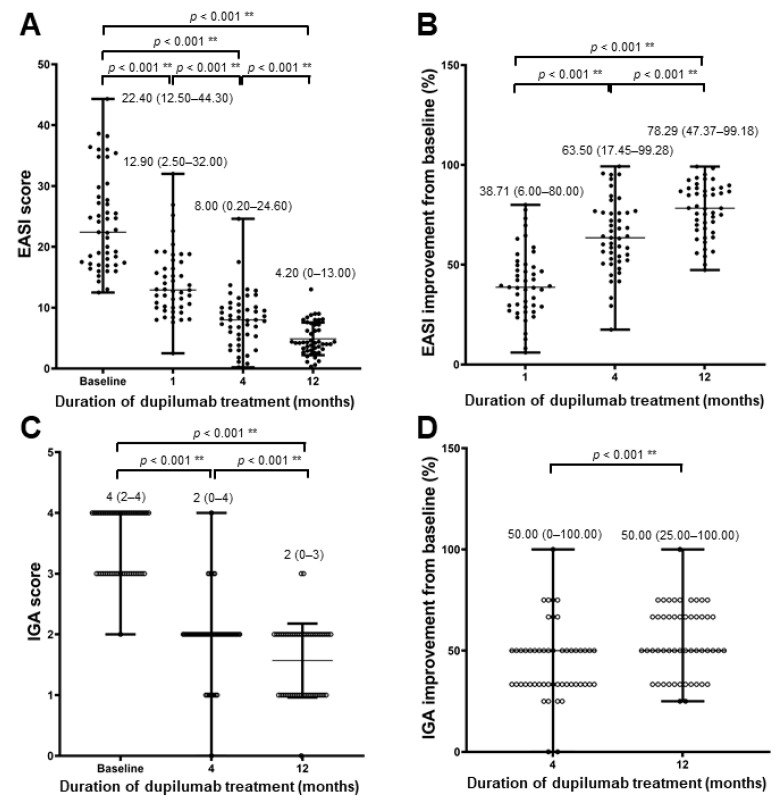
Median EASI scores at baseline, month 4 and month 12. (**A**) Baseline median EASI score was 22.40, which decreased significantly to 12.90 at month 1, 8.00 at month 4, and 4.20 at month 12. (**B**) Median EASI improvement at month 1 was 38.71%, which increased significantly to 63.50% at month 4 and to 78.29% at month 12. (**C**) Median IGA score was 4 (2–4) at baseline, which decreased significantly to 2 (0–4) at month 4 and 2 (0–3) at month 12 (all *p* < 0.001). (**D**) Median IGA improvement at month 4 was 50.00% (0–100.00%), which increased significantly to 50.00% (25.00–100.00%) at month 12. ** *p* < 0.001.

Differences in EASI and IGA scores at baseline, 1st month, 4th month, and 12th month were compared in 51 patients who reached 12 months of treatment. As shown in Figure 1A, the median EASI score was 22.40 at baseline, which decreased significantly to 12.90 at month 1, 8.00 at month 4, and 4.20 at month 12 (all *p* < 0.001). Moreover, the median EASI improvement at month 1 was 38.71%, which increased significantly to 63.50% at month 4 and to 78.29% at month 12 (Figure 1B). Similarly, the median IGA score was 4 (2–4) at baseline, which decreased significantly to 2 (0–4) at month 4 and 2 (0–3) at month 12 (all *p* < 0.001, Figure 1C). The median IGA improvement at month 4 was 50.00% (0–100.00%), which increased significantly to 50.00% (25.00–100.00%) at month 12 (*p* < 0.001, Figure 1D). In addition, among 17 patients with available data, the serum thymus and activation-regulated chemokine (TARC) level significantly decreased at months 1, 2, 3, 4, 5, and 6 compared with that at month 0 (all *p* < 0.05, Figure S2A). Additionally, the serum TARC level at the 4th month significantly decreased in patients who had an EASI-75 at month 4 compared with that at month 0, while no difference was observed between serum TARC level at month 0 and 4 in patients who did not have an EASI-75 (Figure S2B). 

Determination of whether prolonged duration of dupilumab treatment improves EASI-75 and IGA at month 12 is shown in Figure S3A. The proportion of EASI-75 at month 12 increased significantly in patients with EASI score <7 at the 4th month compared with that in patients with EASI score ≥7 at the 4th month. Nevertheless, 51.72% of patients still had EASI score ≥7 at the 4th month, achieving EASI-75 at month 12. The improvement of IGA scores (<2) in these patients was similar but without significant difference (Figure S3B).

### 3.3. EASI-75 Related Factors

**Figure 2:**
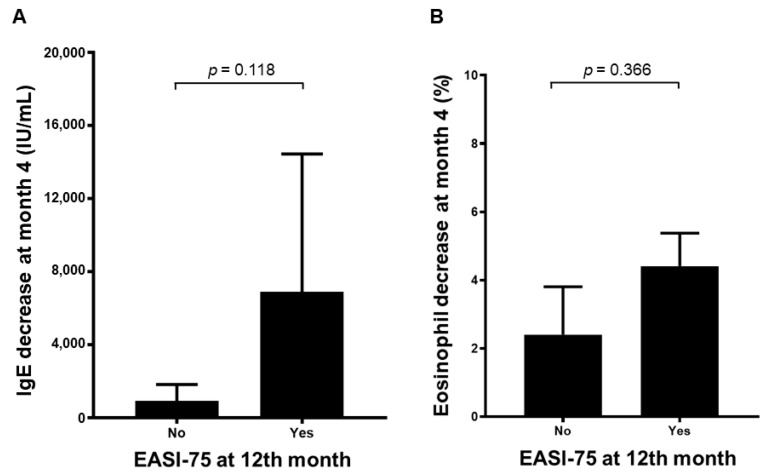
Trends in IgE and eosinophil count. A trend toward a higher decrease in total IgE (**A**) and eosinophil (**B**) at 4th month in patients who had an EASI-75 at the 12th month compared with that in patients who did not have an EASI-75 at the 12th month but without significant difference.

At 4 months of treatment, the significant factors for good response to dupilumab in terms of EASI-75 were identified as younger age, early AD onset, long disease duration until start of dupilumab, high LDH level, and allergic conjunctivitis. Previous use of cyclosporine, azathioprine, or methotrexate and type V phenotype (nummular eczema with round, inflamed sores) were identified as significant factors for poor response to dupilumab in terms of EASI-75 (all *p* < 0.05, Table 3). At 12 months of treatment, prior asthma, early AD onset, and drug interval change to 3 weeks were identified as significant factors for good response to dupilumab in terms of EASI-75, while type III (prurigo with highly pruriginous papules and nodules) and VI (lichenoid pattern with generalized lichenification, excoriations, crusts, and xerosis) phenotypes were identified as significant factors for poor response to dupilumab in terms of EASI-75 (all *p* < 0.05, Table 3). However, gender, weight, prior allergic rhinitis, BSA, baseline eosinophil, and baseline total IgE were not significant factors for response to dupilumab in terms of EASI-75 either at week 4 and at week 16 (all *p* > 0.05, Table 3). Nevertheless, there was a trend toward a higher decrease in total IgE and eosinophil at the 4th month in patients who had an EASI-75 at the 12th month compared with that in patients who did not have an EASI-75 at the 12th month, but there was no significant difference (Figure 2). 

**Figure 3:**
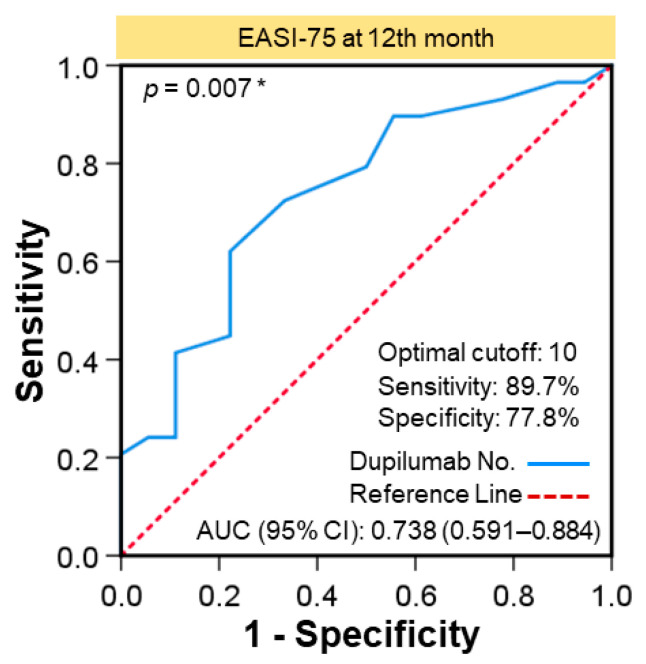
Predictive cutoff value of dupilumab doses to reach EASI-75 at month 12. The optimal differential predictive cutoff value of number of dupilumab doses was 10, as shown by ROC analysis, with AUC of 0.738, sensitivity of 89.7%, and specificity of 77.8% (*p =* 0.007). * *p* < 0.05.

Analysis of the potential number of dupilumab doses as a differential predicted marker of EASI-75 at month 12 is shown in Figure 3. In ROC analysis, the optimal differential predicted cutoff value of the number of dupilumab doses was 10, which yielded an AUC of 0.738, sensitivity of 89.7%, and specificity of 77.8% (*p =* 0.007, Figure 3).

### 3.4. Safety of Dupilumab Treatment

The majority of adverse events were mild in severity, including allergic conjunctivitis (*n* = 27; 24.6%), head and neck redness (*n* = 19; 17.1%), alopecia (*n* = 2; 1.8%), HSV infection (*n* = 2; 1.8%), herpes zoster infection (*n* = 3; 2.7%), and psoriasiform dermatitis (*n* = 2; 0.9%) (Table S2).

For those patients with conjunctivitis, the first event had a mean time of 6–8 weeks until its occurrence. After at least 6 months follow-up, nine patients continued to receive intermittent treatment for conjunctivitis. Conjunctivitis symptoms improved within an average of 2–3 months after the first occurrence of symptoms in 17 patients. Two patients discontinued dupilumab treatment (at 5 months and 12 months) due to intolerance of the symptoms of severe conjunctivitis and corneal ulcer.

For those patients with psoriasiform dermatitis, one patient developed psoriasiform dermatitis after 10 months use of dupilumab and discontinued dupilumab due to lack of further improvement. Another patient developed psoriasiform dermatitis after 5 months use of dupilumab. Symptoms were transient and lasted for 1 month, with symptom improvement after the use of topical steroid.

For those patients with alopecia, one patient developed hair loss after using dupilumab for 4 months and discontinued dupilumab due to progressive alopecia. Another patient who developed alopecia areata after using dupilumab for 11 months experienced symptom improvement after intralesional triamcinolone use.

### 3.5. Specific Cases

Compared to classical AD phenotype, prurigo and nummular AD phenotypes were identified as significant factors for poor response to dupilumab in terms of EASI-75 at month 12. Overall, significant improvement was seen after 4 months of treatment in all AD phenotypes for all the assessed scores as described above, persisting up to month 12. After 12 months of treatment, the median EASI decrease from baseline was 80.78%, 90.15%, 66.36%, 80.59%, 72.55%, 63.32%, and 74.66% in type I, type II, type III, type IV, type V, type VI, and type VII patients, respectively (Table S3).

As shown in Figure S4, a male AD patient with uremic prurigo received treatment with dupilumab 300 mg every 2–3 weeks. After 4 months of treatment, the patient had obvious improvement in AD symptoms. In addition, two male AD patients with hyper-IgE syndrome received treatment with dupilumab 300 mg every 2–3 weeks. After 4 months of treatment, patients also had obvious improvements in AD symptoms (Figure S5).

## 4. Discussion

Dupilumab is the first biological agent approved for treatment of moderate to severe AD, and it offers a new therapeutic approach by specifically targeting key inflammatory pathways in the pathophysiology underlying AD. The present retrospective multiple-center study of a cohort of 111 patients with moderate-to-severe AD reflects the Taiwanese real-world experience in the management of this disease using dupilumab as monotherapy. The efficacy of this agent was evaluated for refractory cases and different AD phenotype, and EASI-75-related factors were identified in patients with moderate-to-severe AD, including those who achieved EASI-50, EASI-75, and IGA < 2 at month 4 after starting dupilumab, persisting up to month 12.

Overall treatment with dupilumab improved signs and symptoms of AD with statistically significant reduction in EASI from baseline at 1, 4, and 12 months follow-up. Further significant differences in EASI scores were still present between 1, 4, and 12 months follow-up, indicating the continuation of dramatic improvement. Compared to previous clinical trials (CHRONOS, EASI-75: 64%) [15], EASI-50 was slightly higher than the efficacy in CHRONOS trials (CHRONOS, EASI-75: 70%). EASI-75 (44.1%) at month 4 was lower than the efficacy in the CHRONOS trials (CHRONOS, EASI-75: 64%) and pooled data (meta-analysis, EASI-75: 59.8), although EASI-50 (86.5%) at month 4 was slightly higher than the efficacy in the two studies (CHRONOS: 78%; meta-analysis: 85.1%). At 12 months follow-up, the overall percentage reduction in EASI score for all patients was 78.3% (EASI-50: 97.9% and EASI-75: 61.7%). The results are in accordance with previously reported results of the long-term efficacy of dupilumab in phase 3 clinical trials (CHRONOS) [15]. In the present study, the effectiveness of dupilumab, which was also evaluated by percentage reduction in EASI score and EASI-75 at month 12, was similar to results obtained from clinical trials of dupilumab in combination with topical corticosteroids (CHRONOS, EASI-75: 64%), while EASI-50 was slightly higher than the efficacy in CHRONOS trials (CHRONOS, EASI-75: 70%). This implies that dupilumab as monotherapy reaches similar efficacy of common practice in the clinical setting as concomitant treatment with topical anti-inflammatory agents.

As described above, the population in the present study had a low frequency of dupilumab response at month 4 compared with previous studies. One possible explanation for the discrepancy is that the Asian AD phenotype differs from the European/American AD phenotype. The Asian AD phenotype combines features of AD and psoriasis with increased TH17 polarization [16], which may cause histologic and clinical differences between races/ethnicities [17]. Another explanation is that the present study included all phenotypes of AD patients, including cases with prurigo or lichenoid phenotypes. Notably, the prurigo phenotype responded more slowly than the other phenotypes after early treatment [13]. Nevertheless, the overall percentage of EASI-75 at month 12 was elevated from 44.1% to 61.7% in all patients. In particular, significant EASI-75 was achieved in over half (51.72%) of AD patients presenting with high EASI (≥7) at month 4, while significant EASI-75 was achieved in 77.78% of AD patients presenting with low EASI (<7) at month 4. These results indicate that continuous treatment can improve the dupilumab response rate in AD patients with or without dupilumab response at month 4.

The incidence and prevalence of AD are increasing steadily, especially in industrialized countries. The diagnostic criteria for this disease in children are basically clinical, including pruritus, a chronic relapsing course, typical distribution, family or personal history of atopy, and onset before 2 years of age [12]. When a skin disease clinically similar to AD occurs in adults without a history of AD or atopic disease, diagnosis can be a challenge. In addition, in adults, the clinical and morphological characteristics of AD are different from those of children. In a recent study, Silvestre et al. [5] revealed the different clinical phenotypes by describing the specific characteristics of adult AD. In the present evaluation of 111 adult patients affected by moderate to severe AD who were treated with dupilumab, 52 patients (52.0%) had adult-onset AD. In previous studies by Hello et al. [18] and Tavecchio et al. [13], the percentage of AD was reported to be 18% and 27% in adults, respectively. The higher percentage in the present study may be related to the fact that we studied a group of select candidates with moderate or severe AD who had received dupilumab. The most common clinical phenotype in the present cohort was the classic phenotype, which manifests as multiple extensive erythematous exudative and lichenoid plaques with flexor side of four limbs and trunk, head and neck, as previously described [13,18]. Tavecchio et al. [13] evaluated the EASI scores of the six AD phenotypes at baseline and found that the generalized inflammatory, lichenoid phenotypes and the erythrodermic phenotype all showed a significantly higher EASI than that of the classic phenotype. In the present study, baseline EASI scores were higher significantly in the erythrodermic phenotype (type IV) compared with that in other phenotypes, indicating that the type IV phenotype along with generalized and lichenoid phenotypes is a noted feature of severe AD. Both prurigo and nummular eczema phenotypes responded more slowly than the other phenotypes at week 4, but after 16 and 52 weeks of treatment, these two phenotypes reached an EASI-75 similar to those of the other phenotypes [13]. In contrast, the present study showed that the prurigo (type III) and lichenoid (type VI) phenotypes had a low proportion of EASI-75 at month 12 compared with those of the classic phenotype (type I) and a low median EASI reduction from baseline to month 12 compared with other phenotypes. Nevertheless, after 12 months of treatment, the median EASI reduction from baseline was still 66.36% and 63.32% for type III and type VI patients, respectively. Interestingly, in the present study, severe AD as the type II (generalized eczema with diffuse erythema) phenotype had a high median EASI reduction (90.15%) from baseline to month 12 compared with those of the other phenotypes. Taken together, these results suggest that dupilumab as monotherapy may offer a beneficial strategy for treatment of all AD phenotypes, especially in the generalized eczema phenotype.

Patients with an early AD onset seemed to respond better to dupilumab at week 4 [13]. Even if at week 16, this association was not confirmed in the present study, early onset showed a certain association (OR *=* 1.7) with response in terms of EASI-75, although results were not statistically significant. In the present study, patients with early AD onset had a significantly better response to dupilumab at month 4 or month 12. Furthermore, patients with prior cyclosporine use had a significantly poor response to dupilumab at month 4, but response to dupilumab was not significantly different from baseline at month 12. A trend of high response to dupilumab at month 4 or month 12 was observed in patients with prior asthma, although the proportion of response to dupilumab at month 4 (*p* = 0.074) was not significantly different from baseline in these patients. A previous study demonstrated that AD patients with prior systemic non-steroidal immunosuppressants (NSISS) (e.g., cyclosporine, methotrexate, azathioprine, or mycophenolate mofetil) use had a lower EASI reduction at week 16 than that in patients without prior NSISS use [19].

AD and asthma share a common genetic and pathogenic basis, and several longitudinal studies have provided evidence for the atopic march from AD to allergic asthma [20]. However, because only a few prospective studies have been conducted that start at children’s births and have sufficiently long follow-up, so little is known about the natural course of AD and the potential succession of atopic phenotypes in childhood. These previous findings emphasize the importance of early AD onset and prior asthma as predictors of early (4 months) and long-term (12 months) response to dupilumab, while prior NSISS use may be considered a predictor of early (4 months) response to dupilumab.

In the present study, a higher number of dupilumab doses was significantly associated with increased response to dupilumab at month 12. Subsequent ROC analysis demonstrated that the optimal cutoff value of dupilumab doses was 10 for differential predictive markers of EASI-75 at month 12, with sensitivity of 89.7% and specificity of 77.8%. Recently, Jang et al. [21] reported that most patients had a two-week interval between doses; however, the efficacy of dupilumab in the two-week interval group was not significantly different from that of the groups with longer intervals (3 weeks and 4 weeks). In contrast, the present study showed that 66 (59.5%) patients received dose interval changes of dupilumab after 4 to 6 months of treatment, with interval changes of 3 weeks, 1 month, 2 months, or 3 months. Drug interval changes to 3 weeks were identified as significant factors for response to dupilumab in terms of EASI-75 at month 12. According to these findings, we suggest that dupilumab may be given ten times initially (dupilumab 300mg every 2–3 weeks) with a drug interval of 3 weeks maintained continuously to achieve a good therapeutic effect at 12 months.

A previous study indicating that the absence of hyper-eosinophilia was a predictive biomarker for response at week 4 suggests a possible role of eosinophils in response to dupilumab [13]. In the present study, a trend of high eosinophil decrease after 4 months of treatment was observed in patients with EASI-75 at month 12 compared with that in patients without EASI-75 at month 12 but without statistical significance. This suggests that the trend may predict early (4 weeks) response to dupilumab. Indeed, inhibition of group 2 innate lymphoid (ILC2) cells and, consequently, eosinophils may be one of the mechanisms of action of dupilumab [22]. However, further experimental and clinical studies are needed to confirm this finding.

In one study, no correlation was found between baseline total IgE and response to dupilumab either at week 4 or at week 16 [13]. Another study showed that the ratio between week 4 and baseline total IgE did not correlate with dupilumab response in terms of EASI-75, even though a slight decrease in total IgE was observed in the first 4 weeks of treatment [23]. In the present study, baseline total IgE was not associated with response to dupilumab at month 4 and month 12. In addition, a decrease in total IgE after 4 months of treatment was also not associated with response to dupilumab at month 12, despite levels of certain biomarkers such as total IgE increase with AD severity, as previously described [23]. However, the real-world data of the present study revealed that baseline total IgE level and IgE decrease at month 4 did not demonstrate any association with response to dupilumab at month 4 (early effect) and month 12 (long-term effect).

In the present study, adverse events were mostly represented by allergic conjunctivitis (27/111, 24.3%) and head and neck redness (19/111, 17.1%), both of which are known to be common side effects of dupilumab [24,25,26]. We observed three cases of herpes simplex virus outbreaks, two cases of alopecia and two cases of psoriasiform dermatitis, which are also reported side effects of dupilumab [26,27,28]. The severity of these adverse events led to drug discontinuation in only three cases—one patient with allergic conjunctivitis and corneal ulcer at month 5 and month 12, one patient with exacerbation of psoriasis, and one patient with alopecia. For allergic conjunctivitis, the proportion of this adverse event in the present population provided similar real-world evidence (22.1%) as noted in a previous study [29]. Most ophthalmologic involvement improved after 3 months of appropriate therapy with ophthalmology. The proportion of head and neck redness as an adverse event in the present population was higher than that in a French cohort study (4.2%) [30]. After receiving common treatment, seven of 42 patients (16.7%) in the French cohort discontinued dupilumab owing to this adverse event [30], but no cases in our population had an adverse event leading to the discontinuation of dupilumab treatment. Both clinical and histopathological characterization suggests dupilumab-induced head and neck redness [31], which may be caused by hypersensitivity to Malassezia species associated with blocking the Th2 pathway [32]. For psoriasiform dermatitis, the previous study reports that the majority of psoriasis showed clearance or near clearance with the use of medium-strength to potent topical corticosteroid ointments and 83% continued use of the dupilumab [33]. In the present study, one case had no improvement of psoriasis after receiving common medication, which led to the discontinuation of dupilumab treatment. In particular, this case had an improvement in psoriasis after replacement treatment with JAK inhibitor, indicating that JAK inhibitor may be a suitable approach for the treatment of psoriasiform dermatitis. In that study, psoriasiform lesions appeared during effective treatment with dupilumab for atopic dermatitis, potentially reflecting a shift toward cutaneous IL-23/Th 17 pathway activation with dupilumab-induced suppression of type 2 immunity [33]. For alopecia, one case developed hair loss after the use of dupilumab for 4 months and subsequently discontinued the use of dupilumab due to progressive hair loss. Another case developed hair loss after 11 months of dupilumab treatment, and the hair loss improved after intralesional triamcinolone acetonide. Nevertheless, none of the adverse events were life threatening in the majority of the above affected patients. The percentage of patients who prematurely discontinued therapy (prior to achieving a desirable therapeutic response) ranged from 6.6 to 9.4% (due to adverse effects, lack of efficacy, or protocol violation). However, the lower rate of discontinuation in our population is likely a result of higher adherence to medical therapy in real life compared to clinical trials [11].

### Limitations

This study has several limitations. First, it was a retrospective cohort study and although the cohort was specially selected to include only patients with AD and the use of dupilumab, selection bias cannot be ruled out. Differential losses to follow-up also may result in bias. In addition, data on potential confounding factors were lacking in the medical records databases. Limited data were available in terms of sample size and follow-up time, which may result in underestimations or overestimations of the efficacy of dupilumab and the reporting of important AEs. Monitoring bias is a concern as investigators may have seen patients more frequently for the purpose of collecting data on dupilumab, which may improve patient adherence. Due to the nature of real-world studies, no uniformity exists between studies regarding previous allowed therapies or concomitant therapies. Furthermore, due to the known ocular AEs, clinicians may be restrictive in prescribing dupilumab to AD patients with severe ocular diseases.

## 5. Conclusions

Study results support the efficacy and safety of dupilumab at month 12 in the treatment of moderate-to-severe AD in the real world, particularly in cases with early AD onset, prior asthma, drug interval change to 3 weeks, or prurigo and lichenoid phenotypes. The optimal number of dupilumab doses for these patients has been defined. The fact that over half of cases with poor dupilumab response at month 4 still achieved improved eczema area and severity index (EASI-75) at month 12 strongly suggests that treatment should not be discontinued until reaching a satisfactory clinical response.

## Figures and Tables

**Table 1:** Baseline demographic and clinical characteristics of adult patients with moderate-to-severe atopic dermatitis.

Variables	Total (*n* = 111)
Age, years, median (range)	30 (18–79)
Gender, *n* (%)	
Male	71 (64.0)
Female	40 (36.0)
Weight, kg, median (range)	66.0 (29.6–129.5)
Prior allergic rhinitis, *n* (%)	78 (70.3)
Prior asthma, *n* (%)	29 (26.1)
BSA%, baseline, median (range)	50 (15–97)
IGA score, baseline, *n* (%)	
3	58 (52.3)
4	53 (47.7)
EASI score, baseline, median (range)	21.9 (10.8–56.3)
<16	18 (16.2)
≧16	93 (83.8)
Onset age, years, median (range)	18 (0–72)
Adult Onset	52/100 (52.0)
Childhood	48/100 (48.0)
Renal Insufficiency	5 (4.5)
Liver disease (HCV, HBV, fatty liver)	4 (3.6)
Hyper IgE syndrome	2 (1.8)
AD phenotype, *n* (%)	
Type I ^a^	50/99 (50.5)
Type II ^b^	6/99 (6.1)
Type III ^c^	7/99 (7.1)
Type IV ^d^	20/99 (20.2)
Type V ^e^	7/99 (7.1)
Type VI ^f^	7/99 (7.1)
Type VII ^g^	2/99 (2.0)
Disease duration until start of dupilumab, years, median (range)	8 (0–43)
Previous use of cyclosporine, azathioprine, or methotrexate, *n* (%)	52 (46.8)
Cyclosporine	38 (34.2)
Azathioprine	25 (22.5)
Methotrexate	21 (18.9)
Laboratory index, baseline, median (range)	
Eosinophil, %	9.1 (0.7–44.0)
LDH, U/L	220 (140–461)
IgE, IU/mL	5000 (17.2–38,700)
Extrinsic AD (IgE > 100)	63/65 (96.9)
Intrinsic AD	2/65 (3.1)

IGA, Investigator’s Global Assessment; BSA, body surface area; EASI, eczema area and severity index; LDH, lactate dehydrogenase; IgE, immunoglobulin E. ^a^ multiple extensive erythematous exudative and lichenoid plaques with flexor side of four limbs and trunk, head and neck; ^b^ generalized eczema with diffuse erythema, predominantly exudative, and crusted eczematous lesions; ^c^ prurigo with highly pruriginous papules and nodules; ^d^ erythroderma with over 90% of the skin surface being red, dry, and lichenified; ^e^ nummular eczema with round, inflamed sores (mainly in, four limbs extensor sides); ^f^ lichenoid pattern with generalized lichenification, excoriations, crusts, and xerosis; ^g^ pattern of follicular eczema.

**Table 2:** Effectiveness of dupilumab in daily practice.

Variables	Total (*n* = 111)
EASI-75, *n* (%)	
Change in score from baseline to 1 month	18/111 (16.2)
Change in score from baseline to 4 months	49/111 (44.1)
Change in score from baseline to 12 months	29/47 (61.7)
EASI-50, *n* (%)	
Change in score from baseline to 1 month	41/111 (36.9)
Change in score from baseline to 4 months	96/111 (86.5)
Change in score from baseline to 12 months	46/47 (97.9)
IGA < 2, *n* (%)	
4 months	29/106 (27.4)
12 months	23/47 (48.9)
EASI reaching MCID after 1 month of treatment	72/111 (64.9)
EASI reaching MCID after 4 months of treatment	104/111 (93.7)
EASI reaching MCID after 12 months of treatment	47/47 (100.0)

EASI, eczema area and severity Index; IGA, Investigator’s Global Assessment; MCID, minimal clinically important difference (EASI improvement from baseline ≥ 6.6), minimal clinically important difference.

**Table 3:** Associations between patients’ characteristics and EASI-75.

	EASI-75 at 4 Months (*n* = 111)		*p*-Value	EASI-75 at 12 Months (*n =* 47)		*p*-Value
Variables	No	Yes		No	Yes	
Age, years, median (range)	34 (18–79)	25 (18–64)	0.005 *	34 (19–76)	28 (19–50)	0.174
Gender/Male, *n* (%)	39 (63.9)	27 (61.4)	0.839	10 (55.6)	22 (75.9)	0.202
Weight, kg, median (range)	68.5 (46.0–103.0)	60.2 (29.6–110.0)	0.077	66.1 (50.0–79.4)	69.8 (46.0–110.0)	0.360
Prior allergic rhinitis, *n* (%)	47 (77.0)	27 (61.4)	0.089	14 (77.8)	25 (86.2)	0.692
Prior asthma, *n* (%)	12 (19.7)	16 (36.4)	0.074	3 (16.7)	14 (48.3)	0.034 *
BSA %, baseline, median (range)	50 (15–90)	50 (20–97)	0.817	40 (25–90)	60 (20–97)	0.216
Onset age, years, median (range)	20 (0–72)	13 (0–51)	0.009 *	20 (8–72)	10 (0.25–46)	0.011 *
AD phenotype, *n* (%)			0.012 *			0.017 *
Type I ^a^	22 (40.0)	26 (65.0)	Ref.	5 (29.4)	16 (61.5)	Ref.
Type II ^b^	4 (7.3)	2 (5.0)	0.413	0 (0)	2 (7.7)	1.000
Type III ^c^	6 (10.9)	1 (2.5)	0.101	3 (17.6)	0 (0)	0.028 *
Type IV ^d^	8 (14.5)	10 (25.0)	1.000	4 (23.5)	5 (19.2)	0.389
Type V ^e^	7 (12.7)	0 (0)	0.010 *	2 (11.8)	3 (11.5)	0.588
Type VI ^f^	6 (10.9)	1 (2.5)	0.101	3 (17.6)	0 (0)	0.028 *
Type VII ^g^	2 (3.6)	0 (0)	0.225	0 (0)	0 (0)	—
Previous use of cyclosporine, azathioprine, or methotrexate, *n* (%)	36 (59.0)	14 (31.8)	0.010 *	12 (66.7)	13 (44.8)	0.229
Cyclosporine	29 (47.5)	9 (20.5)	0.007 *	10 (55.6)	12 (41.4)	0.382
Azathioprine	17 (27.9)	8 (18.2)	0.353	4 (22.2)	4 (13.8)	0.692
Methotrexate	14 (23.0)	5 (11.4)	0.198	6 (33.3)	2 (6.9)	0.052
Disease duration until start of dupilumab, years, median (range)	4 (0–43)	10 (0–34)	0.019 *	3.5 (0–34)	14 (0–34)	0.091
Number of dupilumab doses, median (range)	—	—	—	18 (11–25)	20 (14–28)	0.010 *
Laboratory index, baseline, median (range)						
Eosinophils, %	8.1 (0.7–44.0)	12.1 (2.0–23.8)	0.202	7.7 (2.7–44.0)	12.0 (0.9–31.0)	0.320
LDH, U/L	189 (140–398)	354 (241–445)	0.045 *	198 (198–198)	316 (196–435)	1.000
IgE, IU/mL	5060 (17.2–36,400)	4101 (755–38,700)	0.417	5060 (17.2–25,400)	5000 (755–34,800)	0.707
Atopic/allergic conditions, *n* (%)						
Allergic conjunctivitis	8 (13.1)	19 (43.2)	0.001 *	1 (5.6)	9 (31.0)	0.065
Facial redness	11 (18.3)	9 (20.5)	0.806	1 (5.6)	10 (35.7)	0.032 *
Dry eye	3 (4.9)	6 (13.6)	0.161	1 (5.6)	3 (10.3)	1.000
Drug interval change, *n* (%)	—	—	—	14 (87.5)	18 (62.1)	0.094
3 weeks	—	—	—	2 (14.3)	8 (44.4)	0.036 *
1 month	—	—	—	8 (57.1)	10 (55.6)	—
2 months	—	—	—	3 (21.4)	0 (0)	—
3 months	—	—	—	1 (7.1)	0 (0)	—

BSA, body surface area; EASI, eczema area and severity Index; LDH, lactate dehydrogenase; IgE, immunoglobulin E. * *p* < 0.05. ^a^ multiple extensive erythematous exudative and lichenoid plaques with flexor side of four limbs and trunk, head and neck; ^b^ generalized eczema with diffuse erythema, predominantly exudative, and crusted eczematous lesions; ^c^ prurigo with highly pruriginous papules and nodules; ^d^ erythroderma with over 90% of the skin surface being red, dry, and lichenified; ^e^ nummular eczema with round, inflamed sores (mainly in, four limbs extensor sides); ^f^ lichenoid pattern with generalized lichenification, excoriations, crusts, and xerosis; ^g^ pattern of follicular eczema.

## Data Availability

The datasets used and/or analyzed during the current study are available from the corresponding author on request.

## Supplementary Materials

The following supporting information can be downloaded at: https://www.mdpi.com/article/10.3390/jcm11206209/s1, Table S1. Discontinuation of dupilumab treatment within 12 months follow-up. Table S2. Most common adverse events reported by patients receiving dupilumab. Table S3. EASI decrease from baseline to month 12 in AD phenotype. Figure S1. Patients were stratified into 7 clinical phenotypes by describing the specific characteristics of adult AD. Figure S2. Serum TARC level after dupilumab treatment. Figure S3. Prolonged duration of dupilumab treatment improves EASI-75 and IGA at month 12. Figure S4. Individual AD case with uremic prurigo. Figure S5. Individual AD cases with hyper-IgE syndrome.
