# Supplementary material for: Efficacy of Dupilumab on Different Phenotypes of Adult with Moderate-to-Severe Atopic Dermatitis in Taiwan: A Real-World Study
Source: J Clin Med. 2022 Oct 21;11(20):6209. doi: 10.3390/jcm11206209 (PMC9605350; doi:10.3390/jcm11206209)
Supplement: Supplementary file 1 [file jcm-11-06209-s001.zip › jcm-1939994-supplementary.pdf]

Efficacy of Dupilumab on Different Phenotypes of Adult with Moderate-to-Severe Atopic Dermatitis in Taiwan: A Real-World Study

Figure S1. Patients were stratified into 7 clinical phenotypes by describing the specific characteristics of adult AD. (**Figure S1-A**) Type I, multiple extensive erythematous exudative and lichenoid plaques with flexor side of four limbs and trunk, head and neck. (**Figure S1-B**) Type II, generalized eczema with diffuse erythema, predominantly exudative, and crusted eczematous lesions. (**Figure S1-C**) Type III, prurigo with highly pruriginous papules and nodules. (**Figure S1-D**) Type IV, erythroderma with over 90% of the skin surface being red, dry, and lichenified. (**Figure S1-E**) Type V, nummular eczema with round, inflamed sores (mainly in, four limbs extensor sides). (**Figure S1-F**) Type VI, lichenoid pattern with generlized lichenification, excoriations, crusts, and xerosis. (**Figure S1-G**) Type VII, pattern of follicular eczema.

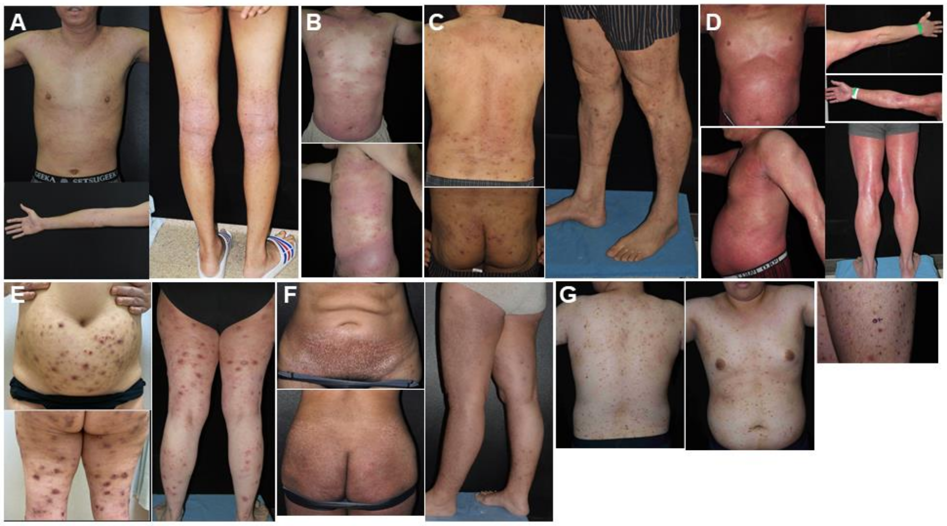

Figure S2. Serum TARC level after dupilumab treatment. (Figure S2-A) Serum TARC level was compared in AD patients (n = 17) during dupilumab treatment. (Figure S2-B) Serum TARC level at month 0 and 4 were compared in AD patients with EASI-75 (n = 13) or non-EASI-75 (n = 4), respectively. HD, healthy donor. * $p < 0.05$. *** $p < 0.001$.

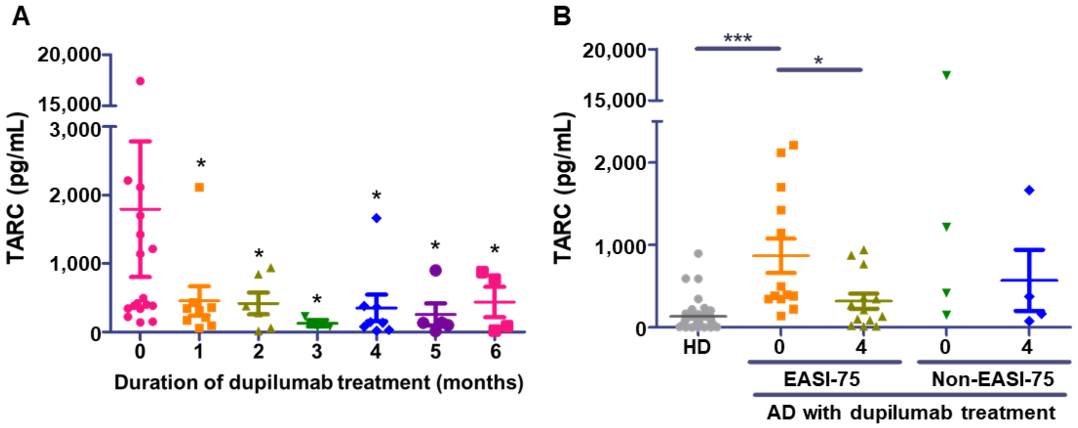

Figure S3. Prolonged duration of dupilumab treatment improves EASI-75 and IGA at month 12. **(Figure S3-A).** The proportion of EASI-75 at month 12 increased significantly in patients with EASI score < 7 at 4th month compared with that in patients with EASI score ≧ 7 at 4th month. Still, 51.72% patients had EASI score ≧ 7 at 4th month, achieving EASI-75 at month 12. (**Figure S3-B**). IGA scores (< 2) had similar improvement, but without significant difference. * $p < 0.05$.

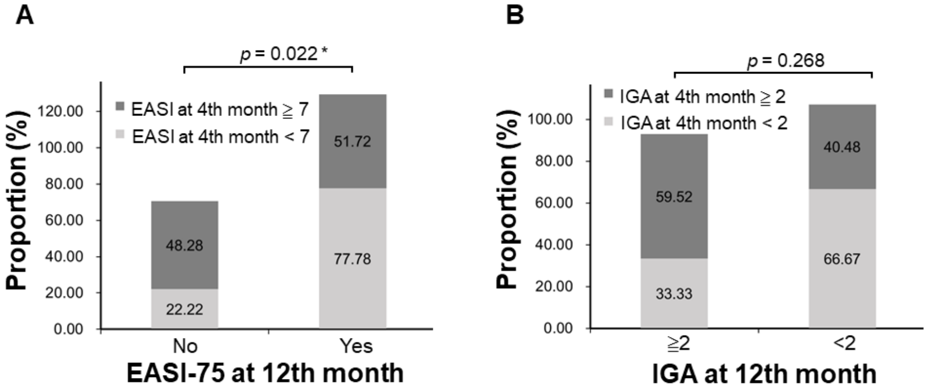

Figure S4. Individual AD case with uremic prurigo. **(Figure S4-A).** A male AD patient with uremic prurigo received treatment with dupilumab 300 mg every 2−3 weeks. **(Figure S4-B).** After 4 months of treatment, the patient showed obvious improvement in AD symptoms.

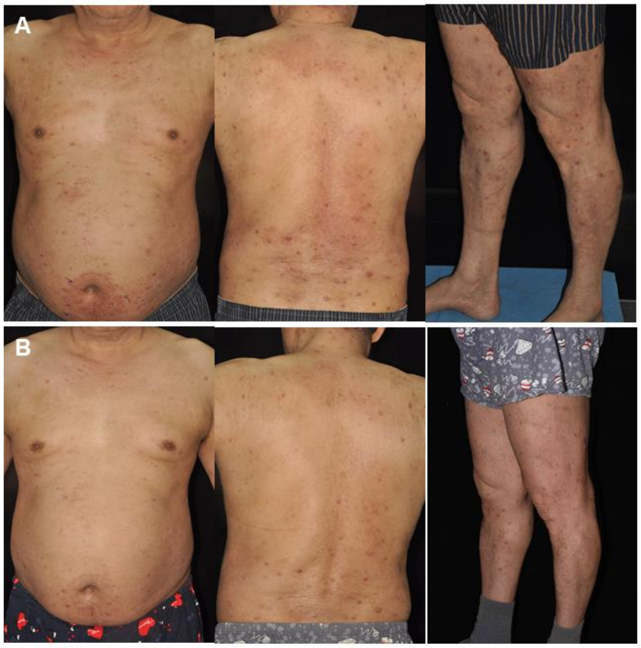

Figure S5. Individual AD case with hyper-IgE syndrome. **(Figure S5-A).** Two male AD patients with hyper-IgE syndrome received treatment with dupilumab 300mg every 2–3 weeks. **(Figure S5-B).** After 4 months of treatment, both patients showed obvious improvements in AD symptoms

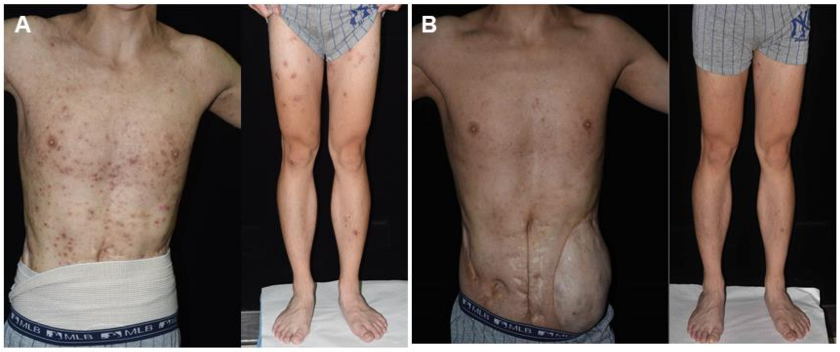

Table S1. Discontinuation of dupilumab treatment within 12 month follow-up.

Reason	n (%)
Partial response, insufficient response	5/18 (27.8)
Moderate to complete clearance of disease	13/18 (72.2)
Ocular symptom	2/18 (11.1)
Economy factor (with good response)	4/18 (22.2)
Exacerbation of psoriasis	1/18 (5.6)
Alopecia	1/18 (5.6)

Table S2. Most common adverse events reported by patients receiving dupilumab.

Adverse event	n (%)
Allergic conjunctivitis	27 (24.3)
Head and neck redness	19 (17.1)
Alopecia	2 (1.8)
HSV infection	2 (1.8)
Psoriasiform dermatitis	2 (1.8)

HSV, herpes simplex virus. Head and neck redness defined as "the occurrence of facial and neck erythema with no particular head and neck involvement at baseline or 50% worsening of the eczema signs from baseline on the head and neck area".

Table S3. EASI decrease from baseline to month 12 in AD phenotype.

	**Phenotype**	**EASI reduction (%), median**
Type I	Lichenoid and exudative plaques with flexor side of four limbs and trunk, head and neck	80.78
Type II	Generalized eczema with diffuse erythema	90.15
TypeIII	Prurigo	66.36
TypeIV	Erythroderma	80.59
TypeV	Nummural eczema	72.55
TypeVI	Lichenoid	63.32
TypeVII	Follicular	74.66
